# From bench to bedside, blade, and back: FAP expression in juvenile angiofibroma. Potential implications for FAPI-PET/CT imaging and targeted therapy?

**DOI:** 10.1007/s00259-025-07468-9

**Published:** 2025-07-22

**Authors:** Lukas Pillong, Caroline Burgard, Florian Rosar, Betül Demirkol, Rafail Ebner, Maximilian Linxweiler, Alessandro Bozzato, Bernhard Schick, Silke Wemmert

**Affiliations:** 1 Department of Otorhinolaryngology, Head and Neck Surgery, Saarland University, 66421 Homburg, Germany; 2 Department of Nuclear Medicine, Saarland University, 66421 Homburg, Germany

**Keywords:** Juvenile angiofibroma, Fibroblast Activation Protein (FAP), FAPI-PET/CT, Epithelial-Mesenchymal Transition (EMT), Tumor biomarker, Non-invasive imaging

## Abstract

**Purpose:**

Juvenile angiofibroma (JA) is a rare, benign fibrovascular tumor that predominantly affects adolescent males. The underlying biological mechanisms remain poorly understood. Fibroblast activation protein (FAP), known for its involvement in tumor invasion, matrix remodeling, and angiogenesis, has been implicated in various malignancies but has not been studied in JA so far.

**Methods:**

We investigated FAP expression in JA samples (*N* = 19) using real-time (RT)-PCR (*N* = 10) and immunohistochemistry (*N* = 18). In addition, Vimentin and PECAM1/CD31 were analyzed to further characterize the tumor microenvironment. For one patient, preoperative FAPI-PET/CT imaging was conducted, and FAP expression was correlated with radiotracer uptake. Postoperative histopathological analyses of the excised tumor were performed to validate the imaging findings.

**Results:**

We found consistent expression of FAP, Vimentin and PECAM1/CD31 in all JA analyzed by RT-PCR. Moreover, substantial intra-and intertumor heterogeneity in FAP protein expression was observed, ranging from negative up to strong positive areas. Vimentin and PECAM1/CD31 showed variable expression patterns consistent with the fibrovascular character of JA. FAPI-PET/CT imaging accurately identified the tumor, with radiotracer uptake closely matching the distribution of FAP expression observed histologically.

**Conclusions:**

This study is the first demonstrating FAP expression in JA and validating its occurrence using FAPI-PET/CT imaging. The strong correlation between FAP expression and radiotracer uptake in FAPI-PET/CT highlights the potential of this imaging modality as a non-invasive diagnostic tool. This will improve diagnosis and is the basis for further investigations of FAP-targeted therapies for JA treatment.

**Supplementary Information:**

The online version contains supplementary material available at 10.1007/s00259-025-07468-9.

## Introduction

Juvenile angiofibroma (JA) is a benign fibrovascular tumor that predominantly affects male adolescents. Recent studies suggest that it originates from persistent neural crest cells [1] at the site of remnants of the first branchial arch near the sphenopalatine foramen [2]. This embryological theory explains the tumor’s specific localization and unique vascular supply.

However, its almost exclusive manifestation in male adolescents suggested a hormonal influence. A study investigating various hormone receptor mRNAs unexpectedly identified luteinizing hormone receptor (LHR) in JAs [3]. Further investigations using in-situ hybridization and immunostaining confirmed LHR expression in JAs associated with pathological blood vessels [4]. As the physiological LH serum levels rise in male puberty, this can explain the sex-specific prevalence of JAs.

Epithelial-mesenchymal transition (EMT), a key process in embryogenesis, may further elucidate the distinct tumor architecture in JAs [1]. Numerous studies support this intriguing theory by identifying various embryological markers, such as laminin alpha 2 [5] and TSHZ1 [6]. Moreover, various growth factors, such as TGF-beta 1 [7] and FGF [8], have been identified, affecting both tumors’ vascular and fibrous components. Given that these are not specific to JAs, potential side effects in other body parts must be considered in therapeutic interventions targeting these growth factors.

In this context, the fibroblast activation protein (FAP) may emerge as an intriguing candidate, particularly intending new therapeutic options in JA treatment. FAP is a membrane-bound serine protease with both dipeptidyl peptidase and endopeptidase activity. While FAP expression is typically low or undetectable in healthy tissue [9, 10], its expression has been implicated not only in multiple malignancies but also in wound healing processes and various diseases such as arthritis [9]. Additionally, fibroblast differentiation [11], extracellular matrix (ECM) remodeling, and also tumor invasion and migration are attributed to FAP. Hence, FAP regulates signaling pathways that promote tumor growth and angiogenesis and was reported to be associated with antitumor immune responses [10].

Consequently, the FAP inhibitor (FAPI) has been effectively utilized in PET/CT diagnostics for a variety of oncological and non-oncological diseases [12, 13]. Hereby, FAPI-PET/CT is particularly valuable for tumor diagnosis, as clinical studies demonstrate its high sensitivity and specificity while providing better image contrast than traditional ^18^F-FDG-PET/CT [12]. The ^68^Ga-FAPI tracer displayed greater sensitivity in identifying primary tumors and metastases across multiple cancer types than ^18^F-FDG-PET/CT. This technology enables significant selective tumor uptake, creating new opportunities for non-invasive tumor characterization [14].

However, the role of FAP in JA has not yet been investigated. In this study, we investigated FAP expression in JA, serving as an interesting and promising target for the diagnosis and treatment of juvenile angiofibroma.

## Materials and methods

### Tumor specimens

This study analyzed tumor tissue samples from 19 male JA patients aged 11 to 24 (average age 15.8) who were diagnosed and underwent surgery at Saarland University Hospital between 2010 and 2024 (Suppl. Table 1). Written informed consent was obtained from all patients, and human tissue was used according to the Code of Ethics of the World Medical Association (Declaration of Helsinki) and approved by the Institutional Review Board (#218/10) at Saarland University.

### Real-time PCR (RT-PCR)

#### RNA isolation and quantification

Total RNA was isolated from snap-frozen tumor tissue (*N* = 11) using the RNeasy Mini Kit (Qiagen) according to the manufacturer’s protocol. RNA concentration was quantified with a Nanodrop spectrophotometer, and 100 ng of total RNA was reverse transcribed using the Superscript IV Vilo kit (Thermo Fisher). RT-PCR reactions were carried out in technical triplicates for each primer pair utilizing TaqMan^®^ assays (FAP: Hs00990791_m1; PECAM1/CD31: Hs01065279_m1; Vimentin: Hs00958111_m1; ß2-microglobulin: Hs00187842_m1; Thermo Fisher) on an ABI StepOne Plus™ instrument (Applied Biosystems^®^ Life Technologies). The thermal profile for RT-PCR included a pre-incubation step at 95 °C for 20 s, followed by 40 cycles of denaturation at 95 °C for 1 s and annealing/extension at 60 °C for 20 s.

#### Normalization and data visualization

The PCR data were normalized using Delta-Ct values calculated relative to the housekeeping gene β2-microglobulin to account for input RNA quantity. To examine the relationships between the gene expression levels of FAP, PECAM1/CD31, and Vimentin, the data were assessed for normality and linearity. FAP, PECAM1/CD31, and Vimentin data showed normal distribution. Linearity occurred only for FAP and PECAM1/CD31, permitting Pearson’s correlation. For FAP-Vimentin and PECAM1/CD31-Vimentin, linearity was lacking, requiring Spearman’s rank correlation as a non-parametric test. The data were visualized using a heatmap to display gene expression levels. A divergent color scale was used, with blue indicating high gene expression and yellow indicating low gene expression.

#### Statistical validation, software and tools

All analyses and visualizations were performed using *R* and *Python*, leveraging key libraries such as *pandas* for data manipulation, *seaborn* and *matplotlib* for visualizations, and *scipy* for statistical analyses.

### Immunohistochemistry

To detect the expression of FAP, Vimentin, and PECAM1/CD31 in the different tissue components of JAs, formalin-fixed, paraffin-embedded samples available from 18 patients and two recurrent samples were utilized for immunohistochemistry. From each sample, 3-micron slides were deparaffinized, and heat-induced antigen retrieval was done in citrate buffer (pH 6.0). Incubation with the specific primary antibodies FAP (1:500, ab53066, Abcam), CD31 (1:40, Dako Agilent), and Vimentin (1:1000, M0725, Dako Agilent) was performed overnight at 4 °C. For negative controls, antibody dilution buffers without primary antibodies were applied. The DAKO Fast Red Kit and DAB Kits (K5005 and K5007, Dako Agilent) were used for detection according to the manufacturer’s instructions. Afterward, the slides were counterstained with Mayer’s Hematoxylin (MHS32, Merck). Two independent observers estimated the percentage and distribution of positive cells in JA stromal and endothelial cells. The overall immunoreactivity was scored using a four-tier scale system based on the rate of positive cells: 0, negative; 1, immunoreactivity in less than 25%; 2, moderate immunoreactivity in less than 75%; and 3, immunoreactivity in 75% or more of the cells.

### ^68^Ga-FAPI-04 PET/CT

^68^Ga-FAPI-04 was labeled as described previously [15]. After injecting 146 MBq of ^68^Ga-FAPI-04 (adapted to the patient’s body weight), PET/CT images were acquired 15 min post-injection with a Biograph 40 mCT PET/CT scanner (Siemens Medical Solutions). The slice thickness was 3 mm, and PET acquisition was performed from the skullcap up to and including the liver at 3 min/bed position. The extended field of view was 21.4 cm (TrueV). PET reconstruction was performed with a three-dimensional OSEM algorithm with three iterations, 24 subsets, Gaussian filtering, and a slice thickness of 5 mm. Decay, scatter, attenuation, and random correction were applied. For anatomical localization and attenuation correction, a low-dose CT scan was performed with an X-ray tube voltage of 120 keV and modulation of the tube current with CARE Dose4D with a reference tube current of 35 mAs. The CT scans were performed with a 512 × 512 matrix, reconstructed with an increment of 3 mm and a slice thickness of 5 mm.

## Results

### Gene expression analysis

RT-PCR analyses of 11 samples from 10 juvenile angiofibroma (JA) patients showed the distinct fibrovascular nature of JA. In all tumors, a heterogeneous expression of PECAM1/CD31 as a marker for vascular components and Vimentin, reflecting the mesenchymal and stromal characteristics of the tumor, was observed. Interestingly, all investigated tumors revealed a pronounced expression of FAP.

### Data visualization

**Fig. 1:**
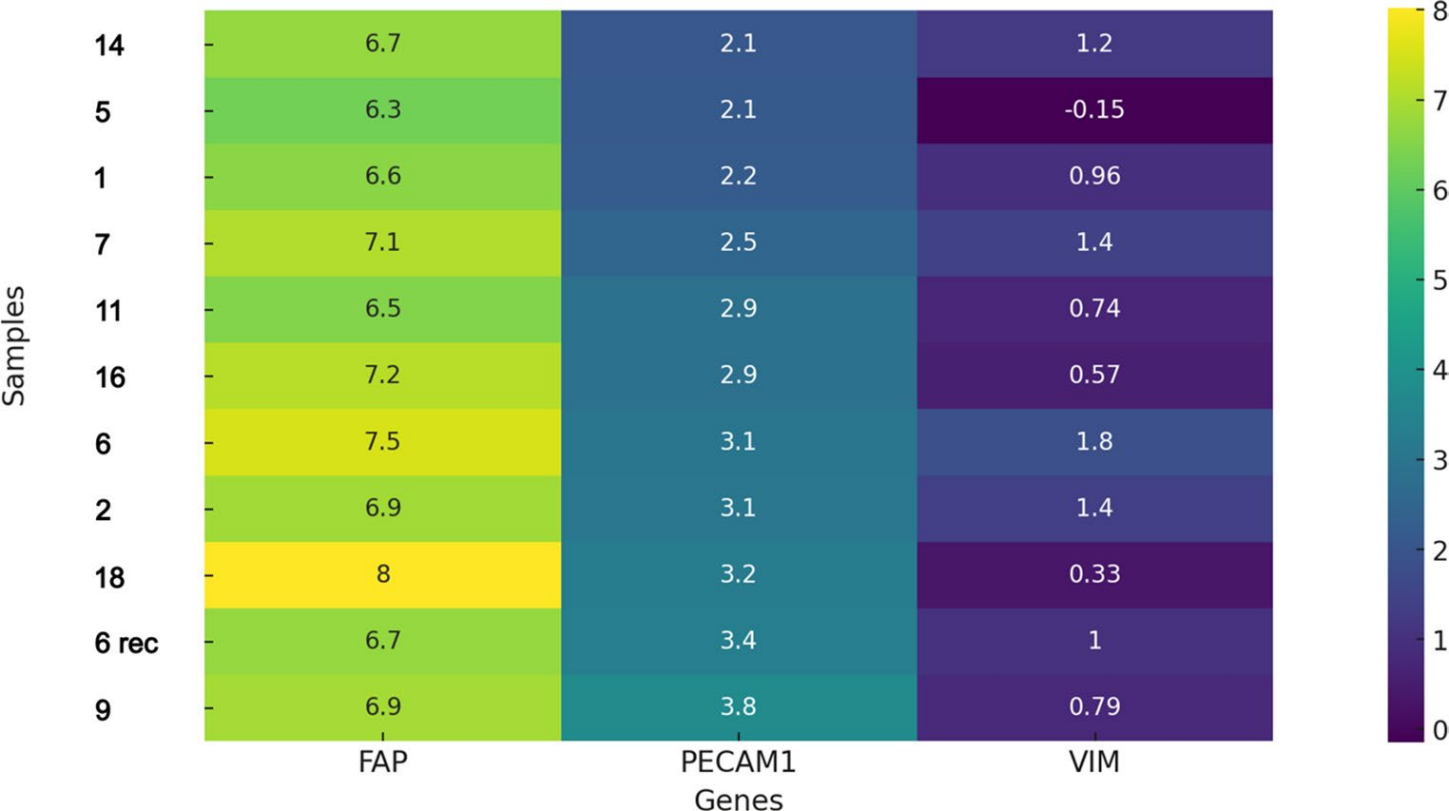
Heatmap visualizing Delta-Ct values for the genes FAP, PECAM1(CD31), and Vimentin (VIM) in juvenile angiofibroma (JA) samples. The color scale ranges from blue (high gene expression) to yellow (low gene expression)

FAP expression was consistently observed in all samples. The heatmap (Fig. 1) displays the samples related to the PECAM1/CD31 expression from high to low. By correlation analysis, a tendency (*r* = 0.52; *p* = 0,08) for a positive relationship between FAP expression and PECAM1/CD31-positive vascular tumor areas was identified. No significant correlation was seen for patient age or grading.

The varying expression levels, also between samples of the same patient (Sample 6), reflect the heterogeneity of JA.

### Immunohistochemistry

**Fig. 2:**
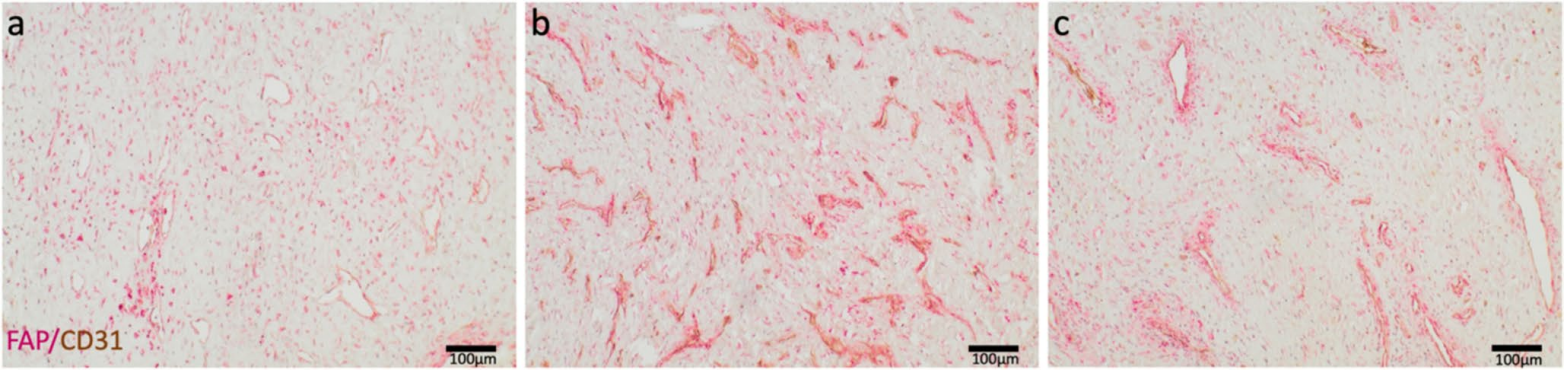
Representative images of FAP (red signal) and CD31 (brown signal) immunohistochemistry of juvenile angiofibroma samples. (**a**) Sample 11, (**b**) Sample 12, and (**c**) Sample 2

Next, immunohistochemical studies validated the relationships indicated by PCR analyses and correlated expression patterns with tumor morphology. A double-staining approach for FAP and CD31 confirmed FAP expression in all JA samples showing FAP-positive stromal cells near CD31-positive vascular regions (Fig. 2) and FAP expression in endothelial CD31-positive cells. As our PCR findings suggested, the varied vascular and stromal components underscore spatial and functional differences within the tumor microenvironment.

### Comparison of tumoral FAP-expression after immunohistochemical staining and ^68^Ga-FAPI-04 uptake

**Fig. 3:**
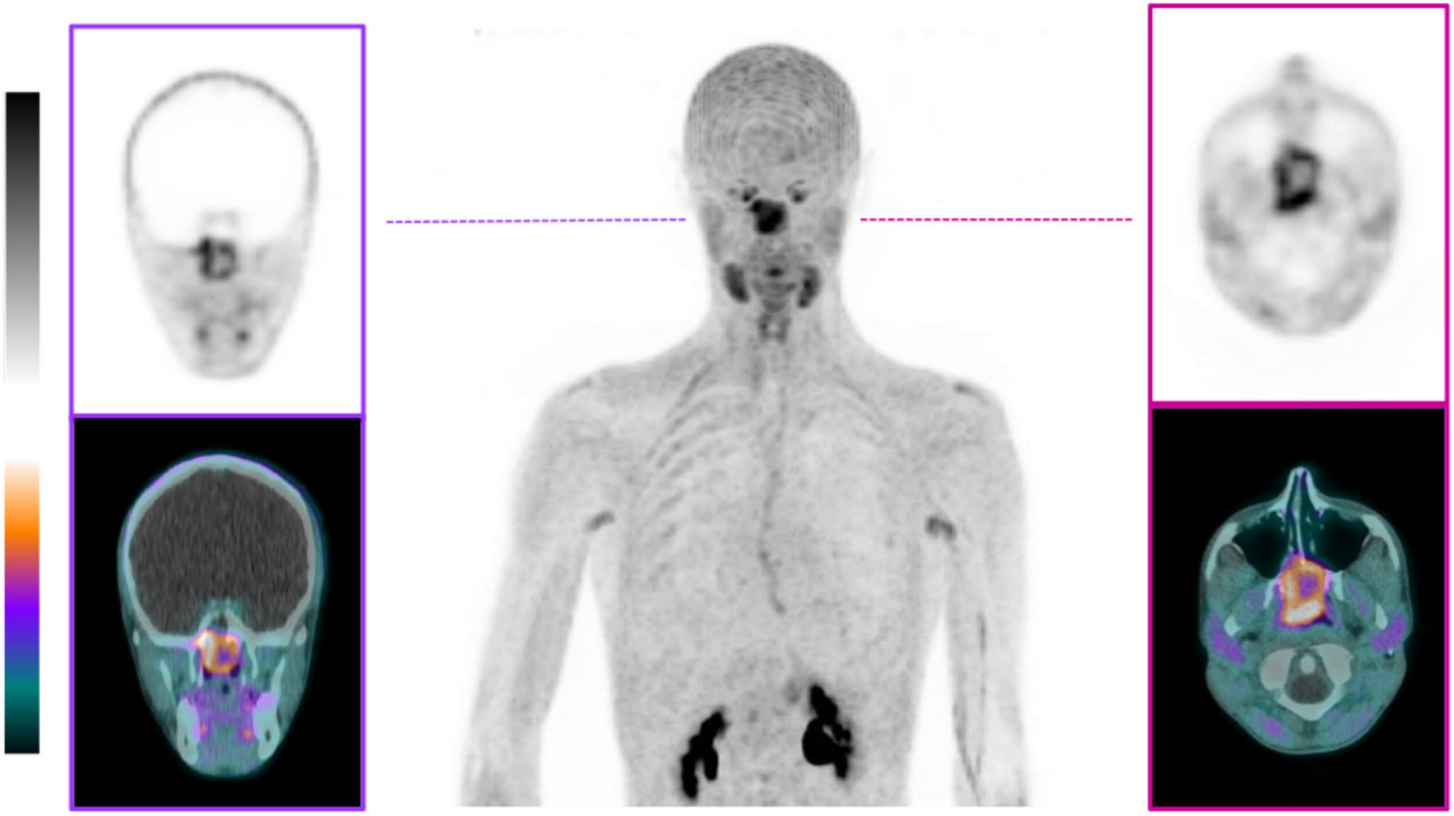
Maximum intensity projection (middle) of ^68^Ga-FAPI-04 PET/CT of an 18-year-old man with histopathologically proven juvenile angiofibroma. Additional coronal (left) and axial (right) reconstructions

Based on the findings from RT-PCR and immunohistochemistry proving FAP expression in juvenile angiofibroma, we pursued the application of ^68^Ga-FAPI-04 PET/CT in a recent suspected case of JA to validate these insights in vivo preoperatively. The FAPI-PET/CT revealed significant uptake in the tumor region. Upon closer analysis of the imaging data, a particularly strong, circular uptake pattern was observed along the entire circumference of the tumor periphery (SUVmax 8.9, SUVpeak 7.1). In contrast, the tumor center exhibited comparatively lower uptake (SUVmax 4.7, SUVpeak 4.5 (Fig. 3)).

### Postoperative correlation of FAPI-PET-uptake distribution with immunohistochemistry

**Fig. 4:**
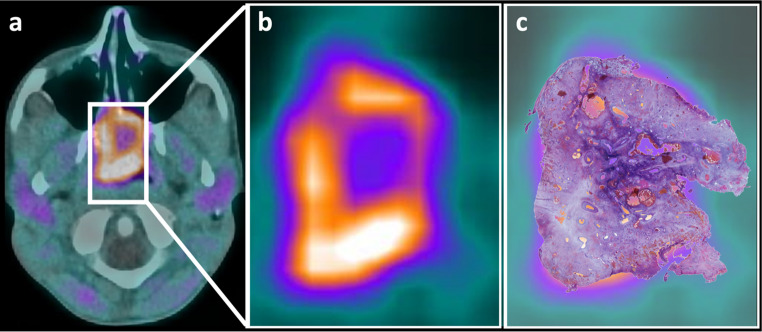
FAPI-PET/CT imaging and corresponding histological projection in juvenile angiofibroma (JA). (**a**) Axial fusion image of FAPI-PET and CT showing the tumor. (**b**) Magnified FAPI-PET view highlighting peripheral tracer uptake and central hypo-uptake in JA. (**c**) Projection of the histological section (Hematoxylin and eosin (HE) staining) from the resected tumor, demonstrating spatial correlation with the imaging findings

The tumor was resected in toto and subsequently processed for immunohistochemical analyses. Interestingly, the uptake characteristics from the FAPI-PET were corroborated postoperatively through immunohistochemical staining of the corresponding specimen (Fig. 4), further underscoring the consistency between preoperative imaging and histological findings.

**Fig. 5:**
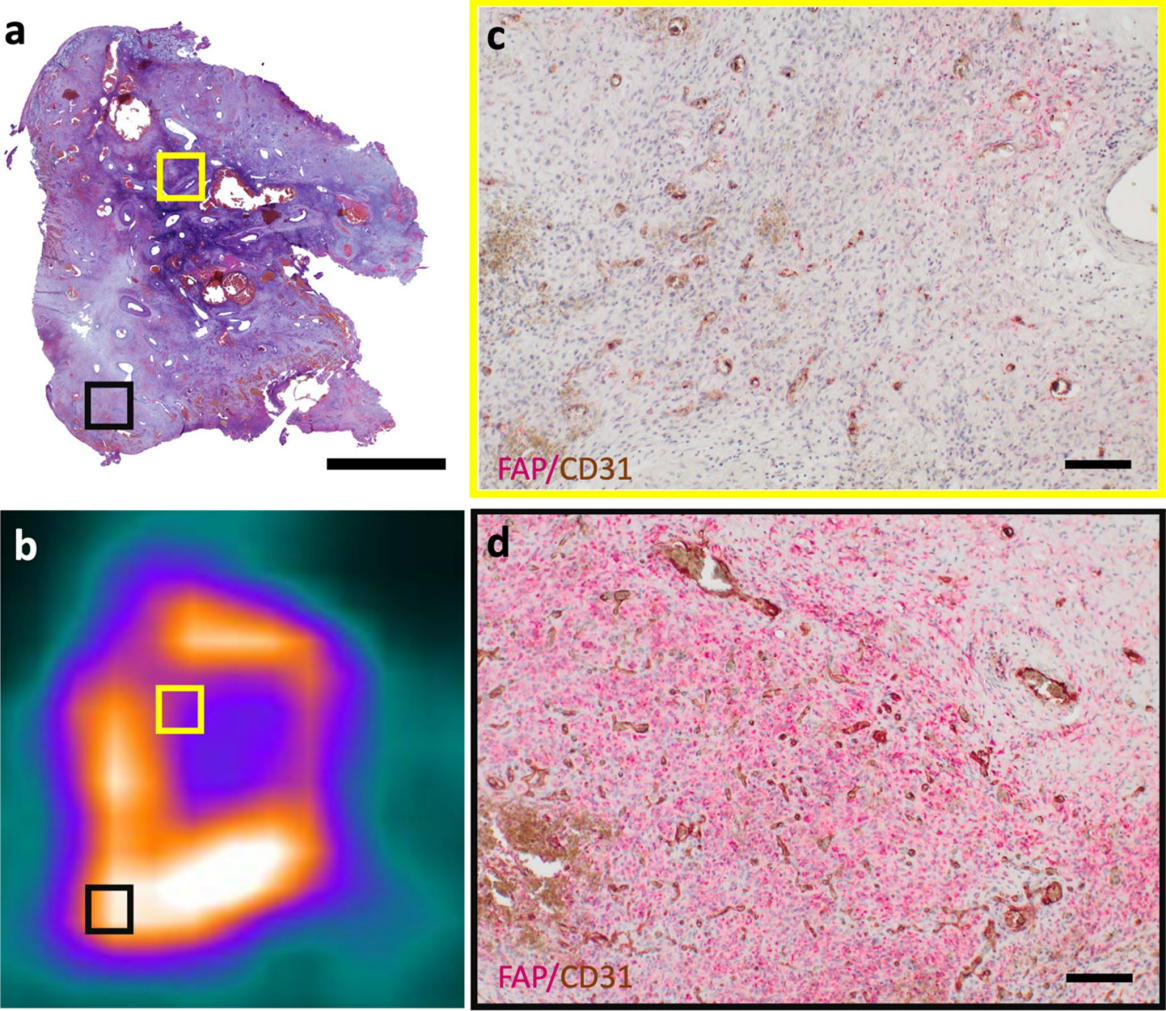
Correlation of histology, FAPI-PET imaging, and immunohistochemistry in juvenile angiofibroma. **a**) HE staining of an axial tumor section (Scale Bar 5.5 mm) matches the FAPI-PET/CT axial slice (**a**). Both images show the tumor periphery (black box) and vascularized center (yellow box) with marked regions. C, d (Scale Bar 100 µm) Immunohistochemical double staining for FAP (red signal) and CD31 (brown signal) from the center (**c**) and periphery (**d**) of the tumor, referenced in A and B. Differential FAP expression is clear, with stronger staining in the periphery (**d**) than in the center (**c**)

Immunohistochemical staining showed pronounced FAP expression in the excised tumor periphery, aligning with tracer-rich areas noted in the FAPI-PET. In contrast, the tumor center had a lower FAP density (Fig. 5).

## Discussion

Due to its distinctive pathophysiological features, JA represents a diagnostic and therapeutic challenge. Current research seeks to understand JA’s pathophysiology to develop new diagnostics and better treatments. As useful targets are lacking, surgical excision remains the gold standard. While the expression of FAP has been well-documented in numerous malignant epithelial tumors [16–23], FAP was not detected in benign epithelial tumors so far [24]. This study is the first to demonstrate FAP expression in JAs. We successfully employed FAPI-PET/CT imaging, providing a direct translational “bench-to-bedside” approach. Our findings offer new biological insights and a promising foundation for innovative diagnostic and therapeutic strategies regarding JAs. Our RT-PCR and immunohistochemical studies confirmed FAP’s role in JA. While there were quantitative differences in FAP expression across samples, PCR showed FAP present in all investigated JAs. Most samples showed moderate FAP expression alongside vimentin and PECAM1/CD31. Individual cases exhibited variations, like higher vimentin with lower FAP and PECAM1/CD31, highlighting tumor morphological heterogeneity and regional adaptability. Additionally, comparisons of samples from the same patient revealed intratumoral heterogeneity based on the expression profile.

Immunohistochemical analyses confirmed heterogeneous FAP distribution, showing varying levels of expression based on stromal and endothelial components. This highlights the potential of targeting FAP in the diagnosis and treatment of JA. FAPI-PET revealed spatial allocation, with higher expression at the tumor periphery and less in the center. Immunohistochemical reactivity against CD31 indicates that FAP is primarily expressed near irregular vascular spaces, the proposed area of tumor origin.

### Role of FAP in EMT and angiogenesis

FAP expression in JA may be explained by the epithelial-mesenchymal transition (EMT), a process that influences tumorigenesis and progression. Previous studies have described EMT as contributing to tumor development [1, 3, 6, 7].

Hereby, EMT is a critical process in tumor biology that facilitates the transition of epithelial into mesenchymal cells. Notably, the perivascular FAP expression in stromal cells correlates with the presence of EMT markers [25]. FAP expression has been observed, furthermore, not only in cells of mesenchymal origin [26–28] but also in transformed neuroectodermal cells [29]. They also contribute to tumor progression, invasion, and metastasis by modifying the extracellular matrix and further releasing pro-angiogenic signals [30–32].

Although the exact role of FAP in JA remains unclear, our findings offer insights into FAP’s potential role in tumor development in JA, indicating it may aid tumor progression via EMT and promote angiogenesis. FAP is primarily expressed in the peripheral tumor tissue of JA, corroborated by studies showing its presence in human endothelial cells, where it influences microvascular reorganization and capillary morphogenesis through enzymatic activity [33–37]. Busek et al. found that FAP is expressed by transformed glioblastoma cells and stromal cells with mesenchymal markers near dysplastic blood vessels. This aligns with perivascular FAP staining in JAs. These mesenchymal cells release pro-angiogenic factors, supporting tumor growth [25]. The angiogenic effects may also arise from its co-expression with MMP-9 as a “pro-angiogenic signaler” [38]. Moreover, an indirect influence of FAP on endothelial cell migration and neovascularization through extracellular matrix reorganization has been described [39]. These findings suggest that FAP may contribute to tumor progression and angiogenesis in JAs. However, its exact pro-angiogenic effects should be explored in further studies. In addition to EMT, stromal activation processes and specific differentiation pathways may also contribute to the expression of FAP in JA. In epithelial tumors, FAP is typically expressed by cancer-associated fibroblasts (CAFs), a heterogeneous population of stromal cells characterized by markers such as FAP, α-smooth muscle actin (SMA), and vimentin. These cells actively participate in extracellular matrix remodeling, immunomodulation, and angiogenesis [40–44]. Activated fibroblasts, called myofibroblasts, also play an important role in wound healing and fibrosis. A hallmark of functional myofibroblasts in this context is the expression of α-SMA, which is associated with increased contractility, ECM production, and secretion of inflammatory mediators [45, 46].

In JA, immunohistochemical studies have shown α-SMA expression in vascular smooth muscle cells and focally in fibrotic-hyalinized stromal areas, suggesting localized myofibroblastic differentiation [47, 48]. Liang et al. identified α-SMA-positive perivascular cells as pericytes. They proposed that these cells are potential precursor cells of the fibrous tumor component, with transitional forms toward myofibroblasts being discussed [49].

A potential functional link between FAP and myofibroblasts is supported by preclinical studies demonstrating FAP as a marker of activated myofibroblasts during wound healing and tissue remodeling [50]. In a myocardial infarction model, FAPI-PET imaging was able to visualize the spatial activation of myofibroblasts, an observation that may represent an interesting parallel to our study. Further phenotypic and functional characterization of stromal cell populations in JA, particularly with regard to potential CAF-like features, therefore appears warranted.

### Clinical validation through FAPI-PET/CT, diagnostic and therapeutic implications

A significant innovation of our study is the clinical validation of the observed FAP expression in JA tissue samples using FAPI PET/CT imaging. An excellent correlation between uptake in ^68^Ga-FAPI-04 PET/CT and the tumoral FAP expression after immunohistochemical FAP-staining with a higher expression in the periphery and a lower amount in the tumor center was shown. This heterogeneous FAP distribution suggests the beginning of EMT in the tumor periphery, associated with possible ECM remodeling.

Usually performed magnetic resonance imaging (MRI) and additional computed tomography (CT) scans reveal, in general, a highly vascularized lesion with a unique growth pattern, including the expansion of the sphenopalatine foramen, extension into the pterygopalatine fossa with anterior bulging of the posterior maxillary sinus wall, as well as extension through other foramina and fissures. Bony destruction may also be observed at the pterygoid plate and along the sphenoid sinus floor. However, expanded pathologies or atypically located JA and uncertainties from limited experience with this rare tumor can complicate radiological differentiation. Radiological assessment is often insufficient in clinical practice, and a biopsy of this highly vascularized tumor has a high risk of severe bleeding. Preferably, an angiography is performed to determine the tumor’s blood supply and identify the typical vascularization pattern before tumor excision. Combined with MRI and CT, this method allows for the diagnosis of JA and gives the potential for simultaneous embolization during angiography.

Our data from retrospective expression and immunohistochemical analyses and its validation through FAPI-PET/CT in a current case suggest that this imaging modality is a sensitive tool for differential diagnosis. FAPI-PET/CT in preoperative diagnostic work could reduce the need for invasive diagnostic procedures, such as angiography, in case of no intended surgery and premature biopsies. This new diagnostic facility offers the chance for follow-up of residual tumors. Hereby, non-intensive uptake in residual JA by FAPI-PET/CT suggests no remarkable growth pattern, enabling a further wait and scan policy.

Surgery can be challenging in highly vascularized tumors. Reducing the amount of vascularity and shrinking the JA size before the tumor is surgically removed is already of great interest. Therapeutic approaches that possess the potential to eradicate tumors completely, thereby making surgical interventions unnecessary, are particularly thrilling. In this context, innovative pharmacological treatment options are of great interest for treating JAs. In other tumor diseases, numerous FAP inhibitors have been investigated, and also theranostic approaches have been explored in nuclear medicine. FAP tracers can be used for targeted radionuclide therapies, specifically destroying tumor cells while sparing healthy tissue. FAP molecules coupled with therapeutic emitters such as Yttrium-90 enable diagnostic and therapeutic applications [12]. First clinical applications with ^90^Y-, ^177^Lu-, ^225^Ac-, and ^153^Sm-FAP tracers showed promising results [12, 14, 51]. FAP-binding peptides, such as FAP-2286, coupled to a radionuclide chelator, have been proposed for imaging using ^68^Ga-FAP-2286 and for therapeutic application using ^177^Lu-FAP-2286 in a wide range of FAP-positive tumors [51, 52].

Moreover, CAR-T cell therapies targeting FAP-expressing cells have also shown promising results in preclinical studies and are becoming an effective treatment method [53–55]. In addition, FAP-targeted monoclonal antibodies are under investigation [56, 57]. Furthermore, small-molecule inhibitors [58], combined with cytostatics [59] or chemotherapeutics [60], showed promising results in Phase II trials.

The identification of FAP in JA opens new diagnostic and treatment possibilities. These could enhance outcomes and lower risks associated with conventional treatments in complex cases.

## Limitations

Although our study is promising and provides novel insights, several limitations must be considered. The heterogeneity of JA may result in pronounced interindividual variability in FAP expression, influenced by clinical factors such as patient age, tumor size, and stage, as well as by fibrotic remodeling processes and EMT activity. This heterogeneity may impact the sensitivity and specificity of FAPI-PET/CT imaging, as well as the efficacy of FAP-targeted therapies. Functional studies investigating the role of FAP in tumor progression and signaling pathway interactions are therefore needed to deepen our understanding of FAPI-PET/CT diagnostics across different patient subgroups and to refine potential therapeutic targets.

Furthermore, increased FAP expression has also been reported in benign conditions [40, 45], which may limit the specificity of FAPI-PET/CT for JA. Comparative analyses with other benign lesions of the nasopharyngeal region are thus required to validate the specificity of this imaging modality. Additionally, although JA is a benign tumor, its locally aggressive growth pattern and imaging characteristics can overlap with those of malignant head and neck tumors, such as nasopharyngeal carcinoma [61–63], and may even be difficult to distinguish from mesenchymal malignancies such as sarcomas [64]. In this context, a recent study demonstrated high FAP expression in various sarcomas [64], underscoring the need for cautious interpretation of FAPI-PET/CT results and thorough differential diagnosis. Therefore, FAPI-PET/CT findings in JA should always be interpreted within the clinical context, particularly given the tumor’s highly characteristic features—such as patient age, sex, and anatomical location—that support a high pretest probability. Similar to conventional angiography, where nonspecific hypervascularization gains diagnostic value only through clinical correlation, FAPI-PET/CT should be assessed in conjunction with clinical and endoscopic findings to ensure appropriate diagnostic specificity.

## Conclusion

To our knowledge, this is the first study demonstrating FAP expression in JA. We demonstrated its diagnostic significance through an integrative, sequential “from-bench-to-bedside” approach by successfully using FAPI-PET/CT as a new diagnostic modality in these tumors. However, further investigations are necessary to exploit clinical experience and the potential of FAP-targeted approaches in clinical practice to optimize diagnosis and therapy in JA.

## Electronic supplementary material

Below is the link to the electronic supplementary material.

Supplementary Material 1

## Data Availability

The datasets generated and/or analyzed during the current study are available from the corresponding author upon reasonable request.

## References

[1] Schick B, Pillong L, Wenzel G, Wemmert S. Neural crest stem cells in juvenile angiofibromas. Int J Mol Sci. 2022;23:1932. 35216046 10.3390/ijms23041932 PMC8875494

[2] Schick B, Urbschat S. New aspects of pathogenesis of juvenile angiofibroma. Hosp Med. 2004;65:269–73. 15176142 10.12968/hosp.2004.65.5.13701

[3] Schick B, Dlugaiczyk J, Wendler O. Expression of sex hormone receptors in juvenile angiofibromas and antiproliferative effects of receptor modulators. Head Neck. 2014;36:1596–603. 23996526 10.1002/hed.23478

[4] Wemmert S, Pyrski M, Pillong L, Linxweiler M, Zufall F, Leinders-Zufall T, et al. Widespread distribution of luteinizing hormone/choriogonadotropin receptor in human juvenile angiofibroma: implications for a Sex-Specific nasal tumor. Cells. 2024;13:1217. 39056799 10.3390/cells13141217 PMC11274802

[5] Starlinger V, Wendler O, Gramann M, Schick > Bernhard. Laminin expression in juvenile angiofibroma indicates vessel’s early developmental stage. Acta Oto-Laryngol. 2007;127:1310–5. 10.1080/00016480701275220 17851944

[6] Schick B, Wemmert S, Willnecker V, Dlugaiczyk J, Nicolai P, Siwiec H, et al. Genome-wide copy number profiling using a 100K SNP array reveals novel disease-related genes BORIS and TSHZ1 in juvenile angiofibroma. Int J Oncol. 2011;39:1143–51. 21874228 10.3892/ijo.2011.1166

[7] Zhang PJ, Weber R, Liang H-H, Pasha TL, LiVolsi VA. Growth factors and receptors in juvenile nasopharyngeal angiofibroma and nasal polyps: an immunohistochemical study. Arch Pathol Lab Med. 2003;127:1480–4. 14567719 10.5858/2003-127-1480-GFARIJ

[8] Mishra A, Mishra SC, Tripathi AM, Pandey A. Clinical correlation of molecular (VEGF, FGF, PDGF, c-Myc, c-Kit, ras, p53) expression in juvenile nasopharyngeal angiofibroma. Eur Arch Oto-Rhino-Laryngol. 2018;275:2719–26. 10.1007/s00405-018-5110-5 30171340

[9] Hamson EJ, Keane FM, Tholen S, Schilling O, Gorrell MD. Understanding fibroblast activation protein (FAP): substrates, activities, expression and targeting for cancer therapy. Proteom Clin Appl. 2014;8:454–63. 10.1002/prca.201300095 24470260

[10] Fitzgerald AA, Weiner LM. The role of fibroblast activation protein in health and malignancy. Cancer Metastasis Rev. 2020;39:783–803. 32601975 10.1007/s10555-020-09909-3 PMC7487063

[11] Lajiness JD, Conway SJ. Origin, development, and differentiation of cardiac fibroblasts. J Mol Cell Cardiol. 2014;70:2–8. 24231799 10.1016/j.yjmcc.2013.11.003 PMC3995835

[12] Mori Y, Dendl K, Cardinale J, Kratochwil C, Giesel FL, Haberkorn U. FAPI PET: fibroblast activation protein inhibitor use in oncologic and nononcologic disease. Radiology. 2023;306:e220749. 36594838 10.1148/radiol.220749

[13] Kratochwil C, Flechsig P, Lindner T, Abderrahim L, Altmann A, Mier W, et al. 68Ga-FAPI PET/CT: tracer uptake in 28 different kinds of Cancer. J Nucl Med. 2019;60:801–5. 30954939 10.2967/jnumed.119.227967 PMC6581228

[14] Zhao L, Chen J, Pang Y, Fu K, Shang Q, Wu H, et al. Fibroblast activation protein-based theranostics in cancer research: A state-of-the-art review. Theranostics. 2022;12:1557–69. 35198057 10.7150/thno.69475 PMC8825585

[15] Lindner T, Loktev A, Altmann A, Giesel F, Kratochwil C, Debus J, et al. Development of Quinoline-Based theranostic ligands for the targeting of fibroblast activation protein. J Nucl Med. 2018;59:1415–22. 29626119 10.2967/jnumed.118.210443

[16] Garin-Chesa P, Old LJ, Rettig WJ. Cell surface glycoprotein of reactive stromal fibroblasts as a potential antibody target in human epithelial cancers. Proc Natl Acad Sci. 1990;87:7235–9. 2402505 10.1073/pnas.87.18.7235 PMC54718

[17] Henry LR, Lee H-O, Lee JS, Klein-Szanto A, Watts P, Ross EA, et al. Clinical implications of fibroblast activation protein in patients with Colon cancer. Clin Cancer Res. 2007;13:1736–41. 17363526 10.1158/1078-0432.CCR-06-1746

[18] Stremenova J, Krepela E, Mares V, Trim J, Dbaly V, Marek J, et al. Expression and enzymatic activity of dipeptidyl peptidase-IV in human astrocytic tumours are associated with tumour grade. Int J Oncol. 2007;31:785–92. 17786309

[19] Hua X, Yu L, Huang X, Liao Z, Xian Q. Expression and role of fibroblast activation protein-alpha in microinvasive breast carcinoma. Diagn Pathol. 2011;6:111. 22067528 10.1186/1746-1596-6-111 PMC3228672

[20] Shi M, Yu D-H, Chen Y, Zhao C-Y, Zhang J, Liu Q-H, et al. Expression of fibroblast activation protein in human pancreatic adenocarcinoma and its clinicopathological significance. World J Gastroenterol. 2012;18:840–6. 22371645 10.3748/wjg.v18.i8.840 PMC3286148

[21] Mentlein R, Hattermann K, Hemion C, Jungbluth AA, Held-Feindt J. Expression and role of the cell surface protease seprase/fibroblast activation protein-α (FAP-α) in astroglial tumors. Biol Chem. 2011;392:199–207. 20707604 10.1515/BC.2010.119

[22] Jia J, Martin TA, Ye L, Jiang WG. FAP-α (Fibroblast activation protein-α) is involved in the control of human breast cancer cell line growth and motility via the FAK pathway. BMC Cell Biol. 2014;15:16. 24885257 10.1186/1471-2121-15-16 PMC4062507

[23] Wikberg ML, Edin S, Lundberg IV, Guelpen BV, Dahlin AM, Rutegård J, et al. High intratumoral expression of fibroblast activation protein (FAP) in colon cancer is associated with poorer patient prognosis. Tumor Biol. 2013;34:1013–20. 10.1007/s13277-012-0638-2 PMC3597266 23328994

[24] Rezaei S, Gharapapagh E, Dabiri S, Heidari P, Aghanejad A. Theranostics in targeting fibroblast activation protein bearing cells: progress and challenges. Life Sci. 2023;329:121970. 37481033 10.1016/j.lfs.2023.121970 PMC10773987

[25] Busek P, Balaziova E, Matrasova I, Hilser M, Tomas R, Syrucek M, et al. Fibroblast activation protein alpha is expressed by transformed and stromal cells and is associated with mesenchymal features in glioblastoma. Tumor Biol. 2016;37:13961–71. 10.1007/s13277-016-5274-9 27492457

[26] Bae S, Park CW, Son HK, Ju HK, Paik D, Jeon C, et al. Fibroblast activation protein α identifies mesenchymal stromal cells from human bone marrow. Br J Haematol. 2008;142:827–30. 18510677 10.1111/j.1365-2141.2008.07241.x

[27] Tran E, Chinnasamy D, Yu Z, Morgan RA, Lee C-CR, Restifo NP, et al. Immune targeting of fibroblast activation protein triggers recognition of multipotent bone marrow stromal cells and cachexia. J Exp Med. 2013;210:1125–35. 23712432 10.1084/jem.20130110 PMC3674706

[28] Chung K-M, Hsu S-C, Chu Y-R, Lin M-Y, Jiaang W-T, Chen R-H, et al. Fibroblast activation protein (FAP) is essential for the migration of bone marrow mesenchymal stem cells through RhoA activation. PLoS ONE. 2014;9:e88772. 24551161 10.1371/journal.pone.0088772 PMC3923824

[29] Rettig WJ, Garin-Chesa P, Healey JH, Su SL, Ozer HL, Schwab M, et al. Regulation and heteromeric structure of the fibroblast activation protein in normal and transformed cells of mesenchymal and neuroectodermal origin. Cancer Res. 1993;53:3327–35. 8391923

[30] Mikheeva SA, Mikheev AM, Petit A, Beyer R, Oxford RG, Khorasani L, et al. TWIST1 promotes invasion through mesenchymal change in human glioblastoma. Mol Cancer. 2010;9:194. 20646316 10.1186/1476-4598-9-194 PMC2920263

[31] Kahounová Z, Kurfürstová D, Bouchal J, Kharaishvili G, Navrátil J, Remšík J, et al. The fibroblast surface markers FAP, anti-fibroblast, and FSP are expressed by cells of epithelial origin and May be altered during epithelial‐to‐mesenchymal transition. Cytom Part A. 2018;93:941–51. 10.1002/cyto.a.23101 28383825

[32] Wang H, Wu Q, Liu Z, Luo X, Fan Y, Liu Y, et al. Downregulation of FAP suppresses cell proliferation and metastasis through PTEN/PI3K/AKT and Ras-ERK signaling in oral squamous cell carcinoma. Cell Death Dis. 2014;5:e1155–1155. 24722280 10.1038/cddis.2014.122 PMC5424105

[33] Aimes R, Zijlstra A, Hooper J, Ogbourne S, Sit M-L, Fuchs S, et al. Endothelial cell Serine proteases expressed during vascular morphogenesis and angiogenesis. Thromb Haemost. 2003;89:561–72. 12624642

[34] Santos AM, Jung J, Aziz N, Kissil JL, Puré E. Targeting fibroblast activation protein inhibits tumor stromagenesis and growth in mice. J Clin Investig. 2009;119:3613–25. 19920354 10.1172/JCI38988 PMC2786791

[35] Gao L-M, Wang F, Zheng Y, Fu Z-Z, Zheng L, Chen L-L. Roles of fibroblast activation protein and hepatocyte growth factor expressions in angiogenesis and metastasis of gastric Cancer. Pathol Oncol Res. 2019;25:369–76. 29134462 10.1007/s12253-017-0359-3

[36] Ghersi G, Zhao Q, Salamone M, Yeh Y, Zucker S, Chen W-T. The protease complex consisting of dipeptidyl peptidase IV and Seprase plays a role in the migration and invasion of human endothelial cells in collagenous matrices. Cancer Res. 2006;66:4652–61. 16651416 10.1158/0008-5472.CAN-05-1245 PMC1457118

[37] Christiansen VJ, Jackson KW, Lee KN, Downs TD, McKee PA. Targeting Inhibition of fibroblast activation Protein-α and Prolyl oligopeptidase activities on cells common to metastatic tumor microenvironments. Neoplasia. 2013;15:348–58. 23555181 10.1593/neo.121850 PMC3612908

[38] Vu TH, Shipley JM, Bergers G, Berger JE, Helms JA, Hanahan D, et al. MMP-9/Gelatinase B is a key regulator of growth plate angiogenesis and apoptosis of hypertrophic chondrocytes. Cell. 1998;93:411–22. 9590175 10.1016/s0092-8674(00)81169-1 PMC2839071

[39] Huang Y, Simms AE, Mazur A, Wang S, León NR, Jones B, et al. Fibroblast activation protein-α promotes tumor growth and invasion of breast cancer cells through non-enzymatic functions. Clin Exp Metastasis. 2011;28:567–79. 21604185 10.1007/s10585-011-9392-x

[40] Dendl K, Koerber SA, Kratochwil C, Cardinale J, Finck R, Dabir M, et al. FAP and FAPI-PET/CT in malignant and Non-Malignant diseases: A perfect symbiosis?? Cancers. 2021;13:4946. 34638433 10.3390/cancers13194946 PMC8508433

[41] Han C, Liu T, Yin R. Biomarkers for cancer-associated fibroblasts. Biomark Res. 2020;8:64. 33292666 10.1186/s40364-020-00245-w PMC7661188

[42] Cords L, Tietscher S, Anzeneder T, Langwieder C, Rees M, de Souza N, et al. Cancer-associated fibroblast classification in single-cell and Spatial proteomics data. Nat Commun. 2023;14:4294. 37463917 10.1038/s41467-023-39762-1 PMC10354071

[43] Pelon F, Bourachot B, Kieffer Y, Magagna I, Mermet-Meillon F, Bonnet I, et al. Cancer-associated fibroblast heterogeneity in axillary lymph nodes drives metastases in breast cancer through complementary mechanisms. Nat Commun. 2020;11:404. 31964880 10.1038/s41467-019-14134-w PMC6972713

[44] Mathieson L, Koppensteiner L, Dorward DA, O’Connor RA, Akram AR. Cancer-associated fibroblasts expressing fibroblast activation protein and Podoplanin in non-small cell lung cancer predict poor clinical outcome. Br J Cancer. 2024;130:1758–69. 38582812 10.1038/s41416-024-02671-1 PMC11130154

[45] Basalova N, Alexandrushkina N, Grigorieva O, Kulebyakina M, Efimenko A. Fibroblast activation protein alpha (FAPα) in fibrosis: beyond a perspective marker for activated stromal cells?? Biomolecules. 2023;13:1718. 38136590 10.3390/biom13121718 PMC10742035

[46] Yang D, Liu J, Qian H, Zhuang Q. Cancer-associated fibroblasts: from basic science to anticancer therapy. Exp Mol Med. 2023;55:1322–32. 37394578 10.1038/s12276-023-01013-0 PMC10394065

[47] Beham A, Kainz J, Stammberger H, Auböck L, Beham-Schmid C. Immunohistochemical and electron microscopical characterization of stromal cells in nasopharyngeal angiofibromas. Eur Arch Oto-Rhino-Laryngol. 1997;254:196–9. 10.1007/BF00879273 9151019

[48] Beham A, Fletcher CDM, Kainz J, Schmid C, Humer U. Nasopharyngeal angiofibroma: an immunohistochemical study of 32 cases. Virchows Arch A. 1993;423:281–5. 10.1007/BF01606891 8236824

[49] Liang J, Yi Z, Lianq P. The nature of juvenile nasopharyngeal angiofibroma. OtolaryngolHead Neck Surg. 2000;123:475–81. 10.1067/mhn.2000.105061 11020189

[50] Hess A, Renko A, Schäfer A, Jung M, Fraccarollo D, Schmitto JD, et al. Spatial FAP expression as detected by 68 Ga-FAPI-46 identifies myofibroblasts beyond the infarct Scar after reperfusion. Mol Imaging Biol. 2025;27:173–83. 40029570 10.1007/s11307-025-01994-6 PMC12062164

[51] Zboralski D, Hoehne A, Bredenbeck A, Schumann A, Nguyen M, Schneider E, et al. Preclinical evaluation of FAP-2286 for fibroblast activation protein targeted radionuclide imaging and therapy. Eur J Nucl Med Mol Imaging. 2022;49:3651–67. 35608703 10.1007/s00259-022-05842-5 PMC9399058

[52] Baum RP, Schuchardt C, Singh A, Chantadisai M, Robiller FC, Zhang J, et al. Feasibility, Biodistribution and Preliminary Dosimetry in Peptide-Targeted Radionuclide Therapy (PTRT) of Diverse Adenocarcinomas using 177Lu-FAP-2286: First-in-Human Results. J Nucl Med. 2021;63:jnumed.120.259192. 10.2967/jnumed.120.259192 PMC8978187 34168013

[53] Shahvali S, Rahiman N, Jaafari MR, Arabi L. Targeting fibroblast activation protein (FAP): advances in CAR-T cell, antibody, and vaccine in cancer immunotherapy. Drug Deliv Transl Res. 2023;13:2041–56. 36840906 10.1007/s13346-023-01308-9

[54] Kakarla S, Chow KK, Mata M, Shaffer DR, Song X-T, Wu M-F, et al. Antitumor effects of chimeric receptor engineered human T cells directed to tumor stroma. Mol Ther. 2013;21:1611–20. 23732988 10.1038/mt.2013.110 PMC3734659

[55] Wang L-CS, Lo A, Scholler J, Sun J, Majumdar RS, Kapoor V, et al. Targeting fibroblast activation protein in tumor stroma with chimeric antigen receptor T cells can inhibit tumor growth and augment host immunity without severe toxicity. Cancer Immunol Res. 2014;2:154–66. 24778279 10.1158/2326-6066.CIR-13-0027 PMC4007316

[56] Chen M, Sheu M-T, Cheng T-L, Roffler SR, Lin S-Y, Chen Y-J, et al. A novel anti-tumor/anti-tumor-associated fibroblast/anti-mPEG tri-specific antibody to maximize the efficacy of mPEGylated nanomedicines against fibroblast-rich solid tumor. Biomater Sci. 2021;10:202–15. 34826322 10.1039/d1bm01218e

[57] Hofheinz R-D, al-Batran S-E, Hartmann F, Hartung G, Jäger D, Renner C, et al. Stromal antigen targeting by a humanised monoclonal antibody: an early phase II trial of Sibrotuzumab in patients with metastatic colorectal Cancer. Oncol Res Treat. 2003;26:44–8. 10.1159/000069863 12624517

[58] Adams S, Miller GT, Jesson MI, Watanabe T, Jones B, Wallner BP. PT-100, a small molecule dipeptidyl peptidase inhibitor, has potent antitumor effects and augments Antibody-Mediated cytotoxicity via a novel immune mechanism. Cancer Res. 2004;64:5471–80. 15289357 10.1158/0008-5472.CAN-04-0447

[59] Eager RM, Cunningham CC, Senzer N, Richards DA, Raju RN, Jones B, et al. Phase II trial of talabostat and docetaxel in advanced Non-small cell lung Cancer. Clin Oncol. 2009;21:464–72. 10.1016/j.clon.2009.04.007 19501491

[60] Eager RM, Cunningham CC, Senzer NN, Stephenson J, Anthony SP, O’Day SJ, et al. Phase II assessment of talabostat and cisplatin in second-line stage IV melanoma. BMC Cancer. 2009;9:263. 19643020 10.1186/1471-2407-9-263 PMC2731782

[61] Mohebbi S, Aghajanpour M. From juvenile nasopharyngeal angiofibroma to nasopharyngeal carcinoma; A rare case report of nasopharyngeal mass. Bull Emerg Trauma. 2019;7:424–6. 31858008 10.29252/beat-070414 PMC6911716

[62] Mishra S, Praveena NM, Panigrahi RG, Gupta YM. Imaging in the diagnosis of juvenile nasopharyngeal angiofibroma. J Clin Imaging Sci. 2013;3:1. 23878770 10.4103/2156-7514.109469 PMC3716018

[63] Makhasana JAS, Kulkarni MA, Vaze S, Shroff AS. Juvenile nasopharyngeal angiofibroma. J Oral Maxillofac Pathol: JOMFP. 2016;20:330–330. 27601836 10.4103/0973-029X.185908 PMC4989574

[64] Koerber SA, Finck R, Dendl K, Uhl M, Lindner T, Kratochwil C, et al. Novel FAP ligands enable improved imaging contrast in sarcoma patients due to FAPI-PET/CT. Eur J Nucl Med Mol Imaging. 2021;48:3918–24. 34018010 10.1007/s00259-021-05374-4 PMC8484190

