# Supplementary material for: From bench to bedside, blade, and back: FAP expression in juvenile angiofibroma. Potential implications for FAPI-PET/CT imaging and targeted therapy?
Source: Eur J Nucl Med Mol Imaging. 2025 Jul 22;53(2):753–62. doi: 10.1007/s00259-025-07468-9 (PMC12830413; doi:10.1007/s00259-025-07468-9)
Supplement: Supplementary file 1 — Supplementary Material 1 [file 259_2025_7468_MOESM1_ESM.docx]

**Supplementale Table 1.** Patient characteristics and immunohistochemical results.

| \| **No.** \| **Case** \| **Age** \| **Grading** \| **RT PCR** \| \| **FAP** \| \| **Vimentin** \| \| --- \| --- \| --- \| --- \| --- \| --- \| --- \| --- \| --- \| \| \| 1 \| 1041 A1 \| 11 \| 3a \| **x** \| \| 0-2 \| \| 3 \| \| 2 \| 1860 R1 \| 12 \| 3a \| **x** \| \| 1-3 \| \| 3 \| \| 3 \| 895 \| 12 \| 3a \|  \| \| 2 \| \| 3 \| \| 4 \| 680 II \| 13 \| 3a \|  \| \| 2 \| \| 3 \| \| 5 \| 1562 2B \| 13 \| rec \| **x** \| \| 3 \| \| 3 \| \| 6 \| 2125 B \| 13 \| unknown \| **x** \| \| 1-2 \| \| 3 \| \| 6rec \| 2253 \| 14 \| rec \| **x** \| \| 1-2 \| \| 3 \| \| 7 \| 1180 B \| 14 \| 3b \| **x** \| \| 1-2 \| \| 2 \| \| 8 \| 896 \| 15 \| 1 \|  \| \| 1-2 \| \| 3 \| \| 9 \| 691 1D \| 15 \| rec \| **x** \| \| 1-2 \| \| 3 \| \| 10 \| 1044-2 \| 15 \| rec \|  \| \| 1-2 \| \| 3 \| \| 11 \| 1563 2A \| 17 \| unknown \| **x** \| \| 2-3 \| \| 3 \| \| 12 \| 1754 C \| 17 \| 3a \|  \| \| 0-3 \| \| 3 \| \| 13 \| 1017-3 \| 17 \| unknown \|  \| \| 1-2 \| \| 3 \| \| 14 \| 2420 \| 18 \| remission \| **x** \| \|  \| \|  \| \| 15* \| 2571 \| 18 \| unknown \|  \| \| 0-3 \| \| 3 \| \| 16 \| 1196 E1 \| 18 \| rec \| **x** \| \| 0-2 \| \| 3 \| \| 16rec \| 1374 \| 19 \| rec \|  \| \| 0-1 \| \| 3 \| \| 17 \| 1925 B \| 20 \| unknown \|  \| \| 2-3 \| \| 3 \| \| 18 \| 2072 II \| 20 \| unknown \| **x** \| \| 1-2 \| \| 3 \| \| 19 \| 1942 \| 24 \| unknown \|  \| \| 1-3 \| \| 3 \| \|  \|  \|  \|  \|  \| \| \|  \|  \| \|  \|  \|  \|  \| \|  \| \|  \|  \| \|  \|  \|  \|  \| \|  \| \|  \|  \| \|  \|  \|  \|  \| \|  \| \|  \|  \| \| rec- recurrence; unk- unknown; n.a.- not available; * FAPI-PET \| \| \| \| \| \| \| \| \| |  |  |  |  | | |  |  | | |  |
| --- | --- | --- | --- | --- | --- | --- | --- | --- | --- | --- | --- | --- | --- | --- | --- | --- | --- | --- | --- | --- | --- | --- | --- | --- | --- | --- | --- | --- | --- | --- | --- | --- | --- | --- | --- | --- | --- | --- | --- | --- | --- | --- | --- | --- | --- | --- | --- | --- | --- | --- | --- | --- | --- | --- | --- | --- | --- | --- | --- | --- | --- | --- | --- | --- | --- | --- | --- | --- | --- | --- | --- | --- | --- | --- | --- | --- | --- | --- | --- | --- | --- | --- | --- | --- | --- | --- | --- | --- | --- | --- | --- | --- | --- | --- | --- | --- | --- | --- | --- | --- | --- | --- | --- | --- | --- | --- | --- | --- | --- | --- | --- | --- | --- | --- | --- | --- | --- | --- | --- | --- | --- | --- | --- | --- | --- | --- | --- | --- | --- | --- | --- | --- | --- | --- | --- | --- | --- | --- | --- | --- | --- | --- | --- | --- | --- | --- | --- | --- | --- | --- | --- | --- | --- | --- | --- | --- | --- | --- | --- | --- | --- | --- | --- | --- | --- | --- | --- | --- | --- | --- | --- | --- | --- | --- | --- | --- | --- | --- | --- | --- | --- | --- | --- | --- | --- | --- | --- | --- | --- | --- | --- | --- | --- | --- | --- | --- | --- | --- | --- | --- | --- | --- | --- | --- | --- | --- | --- | --- | --- | --- | --- | --- | --- | --- | --- | --- | --- | --- | --- | --- | --- | --- | --- | --- | --- | --- | --- | --- | --- | --- | --- | --- | --- | --- | --- | --- | --- | --- | --- | --- | --- | --- | --- | --- | --- | --- | --- | --- | --- | --- | --- | --- | --- | --- |
|  |  |  |  |  |  |  |  |  |  |  |  |
|  |  |  |  |  | | |  |  | | |  |
|  |  |  |  |  | | |  |  | | |  |
|  |  |  |  |  | | |  |  | | |  |
|  |  |  |  |  | | |  |  | | |  |
|  |  |  |  |  | | |  |  | | |  |
|  |  |  |  |  | | |  |  | | |  |
|  |  |  |  |  | | |  |  | | |  |
|  |  |  |  |  | | |  |  | | |  |
|  |  |  |  |  | | |  |  | | |  |
|  |  |  |  |  | | |  |  | | |  |
|  |  |  |  |  | | |  |  | | |  |
|  |  |  |  |  | | |  |  | | |  |
|  |  |  |  |  | | |  |  | | |  |
|  |  |  |  |  | | |  |  | | |  |
|  |  |  |  |  | | |  |  | | |  |
|  |  |  |  |  | | |  |  | | |  |
|  |  |  |  |  | | |  |  | | |  |
|  |  |  |  |  | | |  |  | | |  |
|  |  |  |  |  | | |  |  | | |  |
|  |  |  |  |  | | |  |  | | |  |
|  |  |  |  |  | | |  |  | | |  |
|  |  |  | | |  |  | | |  |  |  |
|  |  |  | | |  |  | | |  |  |  |
|  |  |  | | |  |  | | |  |  |  |
|  |  |  | | |  |  | | |  |  |  |
|  | | | | | | | | | | | |
